# Molecular cloning and characterization of a novel freezing-inducible DREB1/CBF transcription factor gene in boreal plant Iceland poppy (*Papaver nudicaule*)

**DOI:** 10.1590/1678-4685-GMB-2015-0228

**Published:** 2016-07-25

**Authors:** Zhuo Huang, Jiao He, Xiao-Juan Zhong, Han-Du Guo, Si-Han Jin, Xi Li, Ling-Xia Sun

**Affiliations:** 1 College of Landscape Architecture, Sichuan Agricultural University, Wenjiang, Sichuan, China

**Keywords:** DREB1, expression profile, freezing stress, Iceland poppy, transcription factor

## Abstract

DREB1 of the AP2/ERF superfamily plays a key role in the regulation of plant response to low temperatures. In this study, a novel *DREB1*/CBF transcription factor, *PnDREB1*, was isolated from Iceland poppy (*Papaver nudicaule*), a plant adaptive to low temperature environments. It is homologous to the known *DREB1s* of *Arabidopsis* and other plant species. It also shares similar 3D structure, and conserved and functionally important motifs with DREB1s of *Arabidopsis*. The phylogenetic analysis indicated that the AP2 domain of PnDREB1 is similar to those of *Glycine max, Medicago truncatula*, and *M. sativa. PnDREB1* is constitutively expressed in diverse tissues and is increased in roots. qPCR analyses indicated that *PnDREB1* is significantly induced by freezing treatment as well as by abscissic acid. The expression levels induced by freezing treatment were higher in the variety with higher degree of freezing tolerance. These results suggested that PnDREB1 is a novel and functional DREB1 transcription factor involved in freezing response and possibly in other abiotic stresses. Furthermore, the freezing-induction could be suppressed by exogenous gibberellins acid, indicating that *PnDREB1* might play some role in the GA signaling transduction pathway. This study provides a basis for better understanding the roles of *DREB1* in adaption of Iceland poppy to low temperatures.

## Introduction

Abiotic stress conditions, such as drought, high salinity, and cold, have adverse effects on plant growth and production. As sessile organisms, plants have developed a wide spectrum of adaptation strategies to cope with the inevitable challenges of environmental stress. Many aspects of these adaptation processes, including developmental, physiological and biochemical changes, are regulated by stress-responsive gene expression. Transcription factors (TFs) play pivotal roles in gene expression by regulating expression of downstream genes as *trans*-acting elements via specifically binding to *cis*-acting elements in the promoters of target genes. The *cis*- and *trans*-acting elements involved in the transcriptional responses of stress-responsive genes have been previously identified (Yamaguchi-Shinozaki and Shinozaki, 2006).

The APETALA 2/ethylene-responsive element binding factor (AP2/ERF) superfamily is a large group of TF, usually classified to the AP2, RAV, and ERF families (Sakuma *et al.*, 2002; Licausi *et al.*, 2013). The ERF family is further subdivided into ERF and DREB (dehydration-responsive element-binding protein) subfamilies based on different conserved amino acid residues within their respective AP2 domains (Nakano *et al.*, 2006; Lata and Prasad, 2011; Lata *et al.*, 2011). Among these, many members of DREB subfamily are involved in plant abiotic stress responses by regulating gene expression via the *cis*-acting dehydration-responsive element/C-repeat (DRE/CRT, A/GCCGAC) element (Yamaguchi-Shinozaki *et al.*, 2006, Kidokoro *et al.*, 2015) in the promoters of stress responsive genes, such as *COR15A, RD29A*/*COR78*, and *COR6.6* (Stockinger *et al.*, 1997; Liu *et al.*, 1998; Sakuma *et al.*, 2002; Licausi *et al.*, 2013).

The DREB1 subgroup of DREB subfamily are major regulators of cold-stress responses. Three out of the six *DREB1s* of Arabidopsis, *DREB1A/CBF3, DREB1B/CBF1* and *DREB1C/CBF2* are rapidly induced in response to cold stress (Stockinger *et al.*, 1997; Liu *et al.*, 1998; Gilmour *et al.*, 1998; Shinwari *et al.*, 1998). The overexpression of *AtDREB1/CBF* led to up-regulated expression of cold-inducible genes that function in survival at low temperatures, including those encoding late embryogenesis abundant (LEA) proteins and enzymes for sugar metabolism and fatty acid desaturation (Maruyama *et al.*, 2004; Seki *et al.*, 2001; Fowler and Thomashow 2002). Additionally, the expression levels of *DREB1B/CBF1* and *DREB1C/CBF2* are significantly correlated with freezing tolerance (Hannah *et al.*, 2006). Heterologous expression of *DREB1* was capable to improve multiple abiotic stress tolerances in agricultural crops including tobacco (Kasuga *et al.*, 2004), wheat (Pellegrineschi *et al.*, 2004), rice (Ito *et al.*, 2006), *chrysanthemum* (Hong *et al.*, 2006a,b,c; Hong and Kim, 2005), and *Caragana korshinskii* (Wang *et al.*, 2011), etc.

Cold-inducible DREB1/CBF genes have been isolated from numerous dicotyledonous plant species, such as oilseed rape, *Vaccinium myrtillus* (Oakenfull *et al.*, 2013), *Caragana korshinskii* (Wang *et al.*, 2011), *Capsicum annuum* (Hong and Kim, 2005), grape (Xiao *et al.*, 2006), and *chrysanthemum* (Tong *et al.*, 2009), as well as monocotylous plant species, such as wheat (*Triticum aestivum*), rye (*Secale cereale*) (Jaglo *et al.*, 2001), rice (Dubouzet *et al.*, 2003), maize (Qin *et al.*, 2004), etc.

Iceland poppy (*Papaver nudicaule*) is a dicotyledonous and boreal flowering plant, native to subpolar regions of Europe, Asia and North America, and the mountains of Central Asia. It is adapted to low temperature environments and has been widely utilized as ornamental plants because it yields large, papery, bowl-shaped, lightly fragrant flowers supported by hairy, one foot, curved stems among feathery blue-green foliage 1–6 inches long. Previous studies mainly focused on extraction and analyses of its alkaloid (Philipov *et al.*, 2007; Istatkova *et al.*, 2008; Tatsis *et al.*, 2014). However, no attention has been paid on their acclimation to low temperatures. Our previous study investigated the physiological responses and tolerance of four varieties of Iceland poppy to low temperatures (from 3 to –9 °C) (unpublished). To further understand it's low temperature adaptation at molecular level and reveal novel cold responsive genes, we cloned and characterized a new *DREB1* gene member, named *PnDREB1*, from the Iceland poppy variety Champagne Bubbles, which has prominent freezing tolerance among four varieties previously investigated (unpublished). Sequence similarity and phylogenetic relationship to the known DREB1s were comprehensively analyzed, and its spatial expression patterns and responses to freezing stress and phytohormone were also investigated.

## Materials and Methods

### Plant materials and treatments

A variety of Iceland poppy, Champagne Bubbles (CB), was used for gene cloning and expression analyses. Another variety, Wonderland (WL), with lower freezing performance was also used in expression analysis. Seeds were surface-sterilized with hydrogen peroxide solutions and germinated on plates containing the mixture of local soil and nutrient soil (with a ratio of 1:1). The seedlings were maintained in a greenhouse with a relative humidity of 50–70%, 12 h light at 15 °C and 12 h dark at 10 °C. After three or four leaves emerged, the plants were transferred to plastic pots with 15 cm diameter (one plant per pot).

For freezing treatment, the four-month-old plants with uniform growing status were carefully pulled out from the soil. After cleaning the roots with distilled water, the plants were cultured into Hoagland's solution for three days under normal condition and then transferred into an incubator at 0 °C with light. The leaf and root tissues were sampled at 0, 2, 4, 8, 12, 24 h post treatment; For ABA treatment, the plants were treated in 100 Hoagland's solution containing 100 μM ABA (Shan *et al.*, 2007) under normal growth condition and the leaf and root tissues were sampled at 0, 0.5, 1, 2, 4, 8 and 12 h; For gibberellin (GA) treatment, the 80 μM GA_3_ solution containing 0.02% (v/v) polyoxyethylene-sorbitan monolayrate (Tween-20) were evenly sprayed onto the whole plant. Two hours later, the plants were transferred to freezing treatment and leaf tissues were sampled at 0, 0.5, 1, 2, 4, 8 and 12 h (Shan *et al.*, 2007). Each treatment was repeated three times. Samples consisted of equal tissue quantities from 3 individual plants, which were immediately frozen in liquid nitrogen and stored at -80 °C until their use.

### Nucleic acid extraction

Genomic DNA was isolated from leaves of seedlings with the cetytrimethylammonium bromide (CTAB) procedure as reported by Murray and Thompson (1980). Total RNA in various tissues was extracted according to the manual of the TRIZOL Kit (TIANGEN, Beijing). The qualities and quantities of extracted nucleotide were measured by NanoDrop 2000 (Thermo Fisher, USA).

### Amplification of conserved region of *DREB1*

About 5 μg of total RNA was reverse transcribed with oligo18(dT) primer by using single-stranded cDNA Synthesis Kit (TaKaRa Dalian, China) following the manufacturer's introduction. To amplify the conserved region of *DREB1* from Iceland poppy, a pair of degenerate primers, DREB1-F1 and DREB-F2, was designed based on the alignment of nucleotide sequences of AP2 domains of DREB1s of *Arabidopsis, Glycine max, Nicotiana tabacum, Vitis vinifera, Chrysanthemum*, and *Prunus mume* (Table 1).

**Table 1:** Primer sequences for expression level evaluation.

Primer Name	Sequence (5'-3')	Target gene	Expected size (bp)	Usage
DREB1-F2	CGAACAGTTCTCAACAGTTATCATC	*PnDREB1*	400	Semi quantity RT-PCR
DREB1-R2	CTCACTATATTGATAAGTTGGACTC			
actin-F2	TTGGATTCTGGTGATGGTGT	*Actin1*	300	Semi quantity RT-PCR
actin-R2	GAACCTCTGGACAACGGAACC			
actin-F4	ATGCCCTACCACATGCCATC	*Actin1*	86	QPCR
actin-R4	ACCACGCTCCGTCAAGATTT			
ef1-F2	GGAGGCTGCTGAGATGAACA	*EF1*	77	QPCR
ef1-R2	CACGCTCACGTTCAGCCTTA			
DREB-F3	GCTACACCAGAAATGGCTGC	*PnDREB1*	95	QPCR
DREB-R3	CTCCAGACGGAATCAGCGAA			

The Polymerase Chain Reaction (PCR) amplifications were performed in 25 μL reaction volume, consisting of 1 U Ex-Taq DNA polymerase (TaKaRa), 2.5 μL PCR buffer (supplied with *Taq* DNA polymerase), 1 μL cDNA template, 400 pmol of each primer, 1.5 mM MgCl_2_ and 200 μmol of each dNTP. PCR program was conducted as following: 94 °C for 5 min, 30 cycles at 94 °C for 20 s, 56 °C for 20 s, 72 °C for 20 s, followed by 72 °C for 10 min and incubation at 12 °C. Amplified fragments were separated on 1% agarose gels, and purified using agarose gel DNA purified Kit (TIANGEN, Beijing). Purified fragments were ligated onto pEASY-T1 vector (Transgene Beijing). Five positive clones were screened by PCR with M13 universal primers and sequenced on ABI 3730 sequencer (Invitrogen, Shanghai).

### Amplification of 3' and 5' ends of *DREB1*

The Rapid Amplification of cDNA Ends (RACE) technology was employed to obtain 3' and 5' ends of the target gene. To amplify the 3' end of *DREB1* from Iceland poppy, the gene-specific primers 3'RACE-GSP1 and 3'RACE-GSP2 were designed based on the sequence of conserved region of *DREB1* obtained in a previous step (Table 1). Using the cDNA as template, PCR amplifications were performed using primer pair 3'RACE-GSP1 and 3UPM. The composition of the PCR mixture was the same as described above. The PCR was conducted as following program: 94 °C for 5 min, 30 cycles at 94 °C for 30 s, 54 °C for 40 s, 72 °C for 1 min, followed by 72 °C for 10 min and incubation at 12 °C. The resulting solution was 20-fold diluted and 1 μL was used as template in the second round of PCR by primer pair 3'RACE-GSP2 and 3UPM. The reaction mixture and program were the same as the first round of PCR. The final amplified products were also cloned and sequenced as previously described.

The 5' end of DREB1 was obtained by using 5' Full RACE Kit (Takara, Dalian). All reaction mixtures and programs were performed according to the protocols provided by the manufacture. The annealing temperatures for the first and second rounds of PCR amplifications were 55 °C and 53 °C, respectively. The subsequent PCR product separations, purifications, cloning, and sequencing were done as described above. The primers used are listed in Table 1.

### Obtaining full sequences of *DREB1* of the Iceland poppy

The two primers, DREB1-F2 and DREB1-R2, were designed based on the full length cDNA of DREB1 and were subjected to amplification of full cDNA and genomic sequences of DREB1. PCR amplifications were performed in 50 μL reaction volume, consisting of 2U *Taq* HiFi DNA polymerase (TRANSGENE, Beijing) with high fidelity, 5 μL of HiFi buffer (supplied with DNA polymerase), 2 μL cDNA or DNA templates, 200 pmol of dNTP mixture, and 400 pmol of each primer. The PCR program was: 94 °C for 5 min, 10 cycles at 94 °C for 30 s, 49 °C for 30 s, 72 °C for 1 min, 34 cycles at 94 °C for 30 s, 55 °C for 30 s, 72 °C for 1min, and a final extension at 72 °C for 10 min. The resulting products were also gel-separated, purified, cloned, and sequenced.

### Bioinformatics analyses

The deduced protein sequence was predicted by BioEdit (Hall, 1999). The homology modeling of DREB1 protein was performed by SWISS-MODEL with automated mode (Biasini *et al.*, 2014). The model with the highest sequence similarity to the template and highest GMQE and QMEAN4 scores was chosen to predict the three-dimensional structure. Sequence similarity to the known DREB1s was investigated by BLASTP search against the nr protein database available on the website of National Center of Biotechnology Information and The *Arabidopsis* Information Resource (TAIR, http://www.arabidopsis.org).

The motifs in each protein were analyzed by Multiple Em for Motif Elicitation (MEME version 4. 10.1) (Timothy and Charles, 1994). The AP2/ERF domain in each DREB1 was identified by SMART (Letunic *et al.*, 2012) and the corresponding sequence was retrieved. Multiple sequence alignment of amino acids of the AP2/ERF domain was conducted by using MUSCLE (Edgar, 2004) with default options. Motif logo representing the consensus sequence of AP2/ERF domains was drawn by using WebLogo (Crooks *et al.*, 2004). MEGA5.2 software was employed to reconstruct the phylogenetic tree by maximum likelihood method, with 1000 bootstrap replications (Tamura *et al.*, 2011). The Jones-Taylor-Thornton (JTT) model and a discrete Gamma distribution (+G) with 5 rate categories were chosen based on the test model.

### Semi quantitative reverse transcription PCR (RT-PCR)

The spatial expression of *PnDREB1* in petal, pedicel, leaf, petiole, and root were evaluated by semi quantitative RT-PCR. The primers used are listed in Table 1 and *Actin1* was set as internal standard gene. As the sequence of *Actin1* was unknown in Iceland poppy, we amplified and sequenced the *Actin1* using primers (actin-F1 and actin-R1) (Table S1) designed from known sequences of a wide range of plant species (data not shown) (Figure S1). Then, a pair of primers (actin-F2 and actin-R2) was designed for semi quantitative RT-PCR analysis (Table 1). The 1 μL of 10 diluted cDNA reaction mixture was used as template in a 25 μL PCR volume. The PCR programs were: 94 °C for 3 min followed by 6 cycles of 94 °C for 30 s, 55 °C for 30 s, 72 °C for 30 s, and 19 (for *actin1)* or 24 (for *PnDREB1*) cycles of 94 °C for 30 s, 60 °C for 30 s, 72 °C for 30 s, and a final 72 °C for 5 min. The amplifications for two genes were performed simultaneously in the same PCR thermal cycler with three replicates. The amplified products were separated by 1% agarose gel electrophoresis and visualized by ethidium bromide staining.

### Quantitative real-time PCR (qPCR)

The cDNA templates were synthesized as mentioned previously. qPCR reactions were performed with a BioRad CFX system using the iQ SYBR Green supermix kit (Bio-Rad) according to the manufacturer's instructions. PCR procedure was: pre-incubation at 95 °C for 5 min followed by 40 cycles of denaturation at 95 °C for 15 s, annealing at 60 °C for 15 s, and extension at 72 °C for 15 s. The *Actin1* and *elongation factor 1a* (*EF1*) (Long *et al.*, 2010; Liang *et al.*, 2012) were used as internal controls to quantify the relative transcript level. The sequence of *EF1* was firstly obtained as mentioned above. The primers used for qPCR analyses are listed in Table 1. The amplification specificity was checked with a heat-dissociation protocol (melting curves in the 65–95 °C range) as a final step of the PCR. All primer pairs showed a single peak on the melting curve (Figure S2). For each of the independent experiments, the target and internal control were amplified in separate wells in triplicate. The Cq values were determined automatically by BioRad CFX manager 2.1 (BioRad) and the mean Cq of triplicates was used to calculate the relative level of gene expression by using the 2^–ΔΔCT^ method (Livak and Schmittgen, 2001). The final expression data are presented as means from three independent experiments.

### Data analysis

Means and standard deviations (SD) of the expression data were calculated by using SPSS package version 16.0 (SPSS Inc.). Data were analyzed with one-way analysis of variance (ANOVA) and the mean differences were compared by the least significant difference (LSD) test.

## Results

### Cloning of a *DREB1* gene from Iceland poppy

As no genomic resource for the Iceland poppy is available, a pair of degenerate primers, DREB1-F1 and DREB-F1, was designed based on the conserved AP2/ERF domains of *DREB1s* from several dicot species (data not shown). By RT-PCR, a fragment of 204 bp was obtained (Figure S3a) containing a AP2/ERF domain and showing high degree of sequence similarity to known *DREB1/CBFs* (Data not shown). Based on this sequence, further 3'RACE and 5'RACE were performed and a 767 bp and a 470 bp fragment were obtained, respectively (Figure S3b,c). The three fragments were assembled to a 1035 bp sequence. The sequence contains a continuous open reading frame (ORF) with an initiation codon (ATG) and a stop codon (TGA). A pair of primers was further designed to validate the obtained sequences by RT-PCR and genomic PCR (Figure S3c). The sequencing showed identical results as the assembled primer.

### Sequence analyses

**Figure 1:**
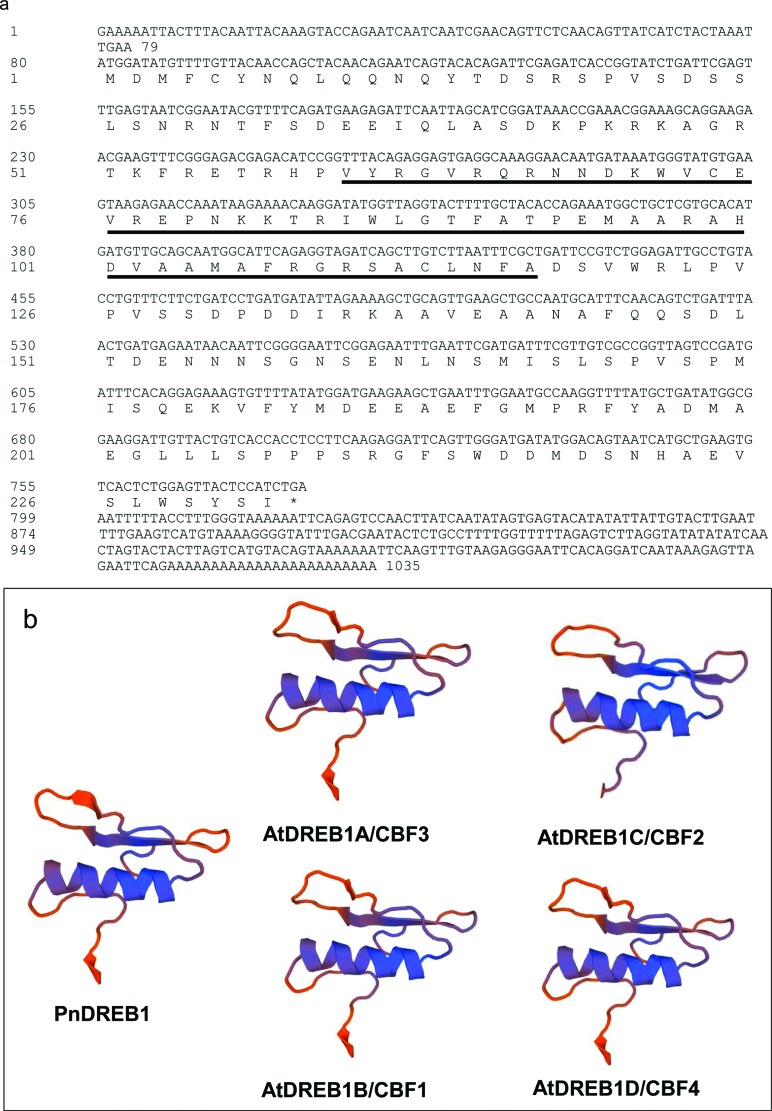
*Sequences of PnDREB1* (a) and comparison of 3D structures of four DREB1 proteins of *Arabidopsis* (b). The AP2/ERF domain is underlined.

The ORF of the obtained sequence is 699 bp long and encodes a deduced protein of 232 amino acids, with 26.3 kDa molecular weight and isoelectric point of 5.33 (Figure 1a). BLAST search against *Arabidopsis* whole genome protein database (TAIR 10) was performed, which indicated that the obtained sequence showed the highest homology to six TFs of A-1 group of *Arabidopsis* DREB subfamily. Homology modeling indicated that the 3D structures of the obtained sequence and the four AtDREB1 proteins contained a conserved AP2/ERF domain with a typical three-dimensional conformation of three antiparallel β-sheets followed by a parallel α-helix (Figure 1b). These results suggested that the obtained gene belongs to DREB1 group of DREB TF subfamily, designated *PnDREB1* (Table 2) (Accession No. KU500634). BLASTP search against the NCBI nr protein database indicated that PnDREB1 shares the highest sequence identity of only 58% (99% query cover and E value= 2e-80) to CBF1 of *Morus alba* var. *multicaulis* (GenBank accession number AFQ59977.1), indicating that *PnDREB1* is a novel *DREB1* gene.

**Table 2:** Homology of PnDREB1 to DREB1s in *Arabidopsis*.

Gene ID	Description	Function	E value
AT5G51990.1	DREB1D/CBF4	Response to drought stress and ABA	2e-50
AT4G25470.1	DREB1C/CBF2	Response to low temperature and circadian rhythm	4e-46
AT4G25490.1	DREB1B/CBF1	Response to low temperature	2e-45
AT4G25480.1	DREB1A/CBF3	Response to low temperature	2e-44
AT1G12610.1	DREB/DDF1	Induce GA biosynthesis under salt stress	4e-38
AT1G63030.1	DREB/DDF2	Reduce ABA biosynthesis by overexpression	5e-37

**Figure 2:**
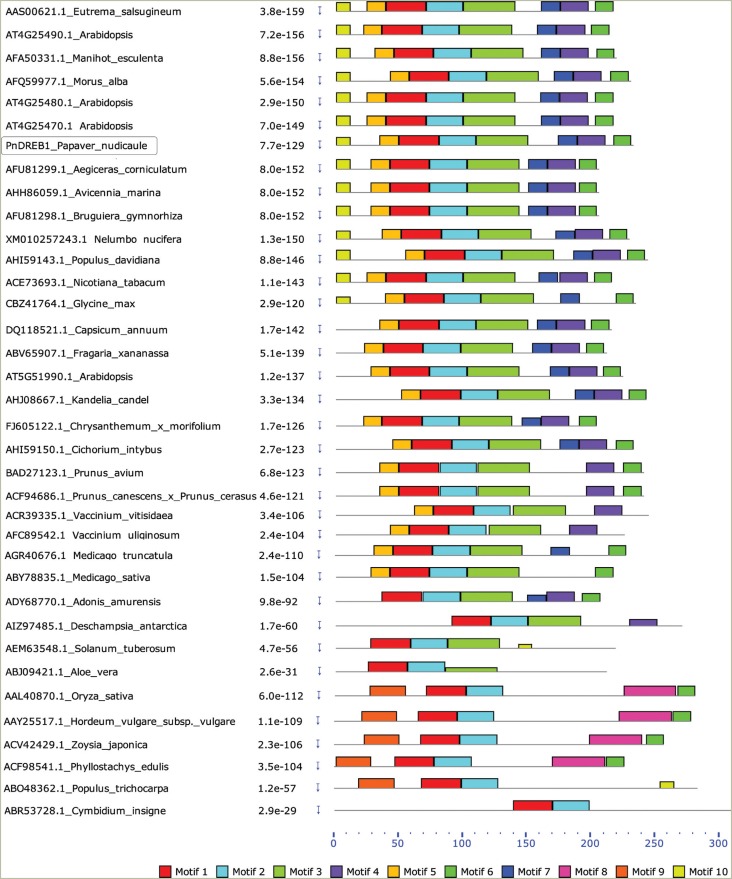
Comparison of protein motifs of 35 DREB1s from diverse dicot and monocot species. The PnDREB1 is boxed.

To evaluate the structural similarity, motif identification and comparison were performed between the PnDREB1 and 35 known DREB1 from 33 different dicots or monocots species (Figure 2). PnDREB1 contains eight motifs, which are similar to those of 12 DREB1s from 10 species, such as *Arabidopsis, M. alba, Manihot esculenta, Avicennia marina*, etc. Motif 1 and motif 2 are shared by all DREB1 proteins, covering the whole AP2/ERF domain. Motifs 3~7 are also common, present in ~79.5% to ~83.3% of the DREB1s analyzed, indicating that they might be functionally important to the DREB1s.

### Comparison of AP2/ERF domain and phylogenetic analysis

**Figure 3:**
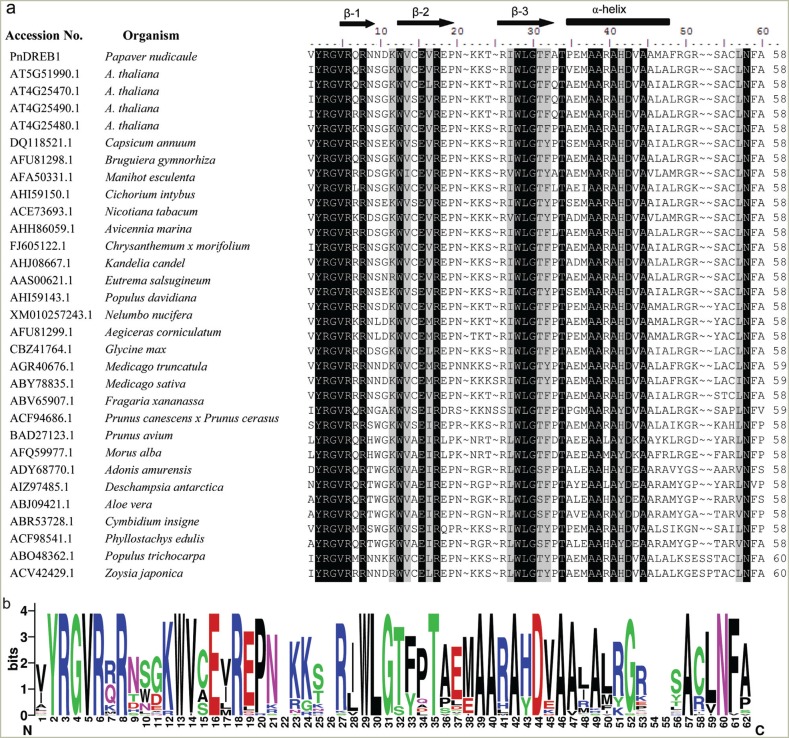
Comparison of deduced amino acid sequences of AP2/ERF domain of 35 DREB1s from diverse dicot and monocot species. a, multiple alignment of amino acid sequences of AP2/ERF domain. Black shading indicates identical residues; gray shading indicates highly conserved residues. b, Motif logo drawn based on the multiple alignment of amino acid sequences of AP2/ERF domain. The overall height of the stack indicates the sequence conservation at that position, while the height of symbols within the stack indicates the relative frequency of each amino at that position.

The AP2/ERF domain sequences were retrieved from the 36 DREB1s as described above. Most of the sequences are composed by 58 residues, with three exceptions, and 2 peptides containing 59 and 60 residues. The multiple sequence alignment showed that the AP2/ERF domain of PnDREB1 is highly homologous to other 35 DREB1s from divergent species (Figure 3a). A total of 19 amino acids are identical among 36 proteins, including motif YRGVR and WLG, and some other residues, such as Arg-8, Trp-13, etc. These conserved residues mainly lie in the regions comprising three β-sheets and one α-helix, which are structurally important. The residues outside these regions are rather divergent. The drawn domain logo showed the variability and conservation of each residue in AP2/ERF domain of DREB1s (Figure 3b).

**Figure 4:**
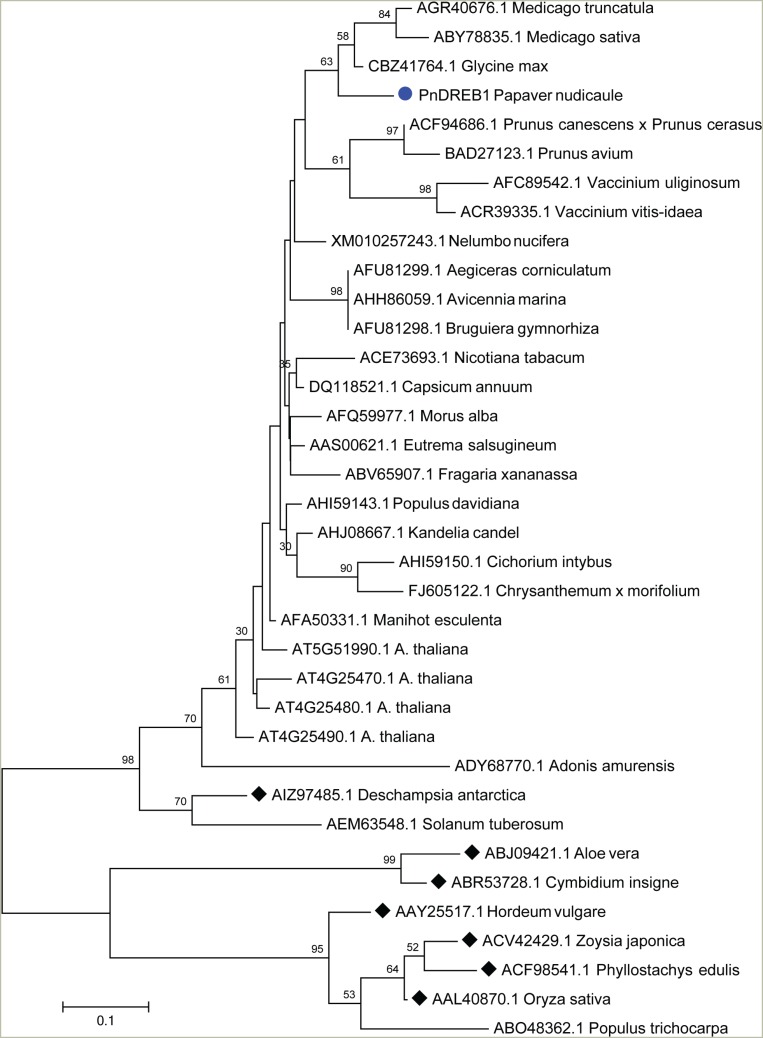
Phylogenetic tree based on the deduced amino acid sequences of AP2/ERF domain of 35 DREB1s. The tree is constructed by maximum likelihood method with 1000 bootstrap replications. Before tree reconstruction, a model test was performed. The model with the lowest BIC scores (Bayesian Information Criterion), the Jones-Taylor-Thornton (JTT) model with parameters of Gamma distribution (+G) with 5 rate categories for Rates and Patterns were chosen. Diamonds indicate the monocot species.

The phylogenetic tree constructed based on the alignment of amino acid sequences of AP2/ERF domain (Figure 4) showed that PnDREB1 was clustered with DREB1s of *G. max*, and species of *Medicago, Prunus*, and *Vaccinium*, and significantly separated from those of monocots.

### Spatial expression patterns, freezing and phytohormone-induced responses

**Figure 5:**
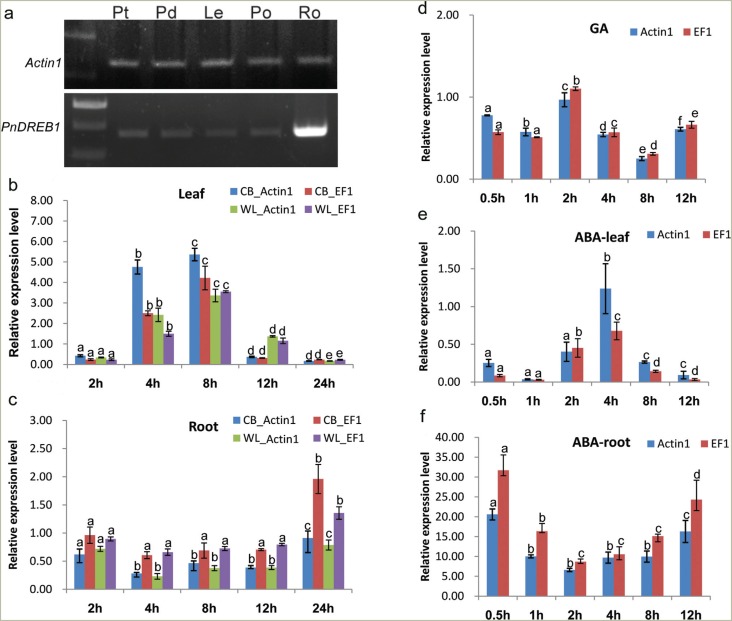
Expression profiles of *PnDREB1*. (a), Semi quantitative RT-PCR analysis of spatial expression patterns in petal (Pt), pedicel (Pd), leaf (Le), petiole (Po), and root (Ro); b and c) show qPCR analysis results; relative expression levels of *PnDREB1* at different time points (x-axis) of freezing treatment in leaf and root, respectively, in fold-change (y-axis); d) qPCR analysis results showing changes of relative expression levels of gibberellins acid- (GA_3_, 80 μM) treated plants under same treatment as those of b); e and f) fold changes of relative expression levels under treatment of 100 μM abscisic acid (ABA). CB, Champagne Bubbles; WL, Wonderland. Data are shown as means ± SD (n = 3). *Actin1* and *EF1* were used as internal controls. Different lowercase letters on rectangular columns indicate significant differences to that of previous time point (P < 0.05).

The spatial expression of *PnDREB1* indicated that, under normal conditions, the expression of *PnDREB1* could be detected in all analyzed tissues, including petal, pedicel, leaf, petiole, and root (Figure 5a). The root exhibited higher expression levels than other tissues.

We further investigated the dynamic changes of *PnDREB1* expression levels under freezing treatment (0 °C) by qPCR. In leaves, the expression level of *PnDREB1* was very low at beginning of the treatment (Figure 5b). After 2h, the expression level significantly increased and reached a peak at 8 h; after 12 h, the expression decreased to similar levels as the initial stage of treatment. In roots, the expression level was slightly higher than those in leaves at the initial stage. After 2 h, the level decreased and remained low until 12 h, when it increased to a slightly higher levels than those before treatment (Figure 5c). We also evaluated the expression levels in another Iceland poppy variety (WL) with lower freezing tolerance. WL showed similar patterns in roots. However, in leaves, it increased slower and exhibited a significantly lower peak expression than CB (Figure 5b).

The responses of *PnDREB1* to the phytohormones gibberellic acid (GA) and abscisic acid (ABA) were investigated by qPCR. Under freezing temperature, the GA_3_-treated plants exhibited lower expression levels compared to non-GA_3_-treated plants (Figure 5d). The ABA treatment was performed under hydroponic growth condition. In leaves, the expression level of *PnDREB1* decreased to the lowest level at 1 h; one hour later, it gradually increased and reached a peak at 4 h; at 12 h, it decreased to a similar level as 1 h. At all time points, levels were lower than that of the control (untreated) (Figure 5e). In roots, the expression level intensely increased ~26-fold that of control within 0.5 h. After that, it was down-regulated to the lowest level of about ~7-fold at 2 h and then was up-regulated again ~20-fold at 12 h (Figure 5f).

## Discussion

*DREB1* has been characterized as an important regulator of cold response among a spectrum of plant species. In the present study, the cDNA and genomic sequences of a novel DREB1 TF, *PnDREB1*, with a high sequence similarity and similar predicted 3D structure to DREB1s of *Arabidopsis*, was isolated from the boreal ornamental plant Iceland poppy. Phylogenetic analysis indicated that the AP2 domain of PnDREB1 is close to those of *G. max*, and *Medicago, Prunus*, and *Vaccinium* species.

A motif is a pattern common to a set of nucleic or amino acid subsequences which share some biological property (Timothy and Charles, 1994). Thus, the motif compositions and distributions among a set of sequences reflect, to a certain extent, the structural and functional similarity. We compared the motifs of PnDREB1 to 35 known DREB1s from 33 species (Figure 2 and Figure S4). All shared high conserved AP2/ERF domain, in which 19 residues are conserved in ~95% of DREB1s. Previous studies showed that the 14th valine (V14) and 19th glutamic acid (E19), especially the former, of the AP2/ERF domain, are conserved among the DREB protein (Liu *et al.*, 1998). They are distinguished from alanine and aspartic acid of ERF protein and are important for its binding specificity (Sakuma *et al.*, 2002). PnDERB1 contained the same conserved V14 and E19 at these two positions, indicating that it might possess similar binding patterns as DREB1s of *Arabidopsis* to DRE/CRT motif in the promoter of some downstream stress-induced genes.

Nakano *et al.* (2006) reported that some motifs outside the AP2/ERF domain are also conserved for DREB1 proteins. Motif CMIII-1 is common for DREB1s; CMIII-2 and CMIII-4 are conserved in C-terminal region, and CMIII-4, also known as LWSY motifs, is conserved in rice and *Arabidopsis* and has been shown to function as a transactivation domain (Wang *et al.*, 2005). The CMIII-3, separated by AP2/ERF domain, is also conserved and was reported in other studies (Jaglo *et al.*, 2001; Haake *et al.*, 2002). Despite of different methods used for motif identification, PnDREB1 was found to contain all of these motifs: motif 3 covers CMIII-1; motif 5 and part of motif 3 is equivalent to CMIII-3; the adjacent motif 7 and motif 4 are approximate to the CMIII-2, and CMIII-4 is involved in motif 6. These results indicated that PnDREB1 might be an active stress-induced DREB1 protein.

Our further investigation of dynamic expression changes under freezing treatment showed that *PnDREB1* was induced by freezing both in leaves and roots though in different patterns. The expression level in leaves was quickly upregulated and reached peak level at 8h. These results are similar to some reports in other species (Stockinger *et al.*, 1997; Liu *et al.*, 1998; Qin *et al.*, 2004; Huang *et al.*, 2007; Shan *et al.*, 2007; Kidokoro *et al.*, 2015). Interestingly, freezing-induced expression in leaves could be suppressed by exogenous GA_3_. This phenomenon was also found in cotton, indicating that it may play an important role in GA signaling (Shan *et al.*, 2007). Our comparative analysis also indicated that expression of *PnDREB1* in the CB variety with high freezing tolerance increases faster and accumulates to higher levels than those in WL variety with lower freezing tolerance. The difference in freezing inductive accumulation of *PnDREB1* transcription level might partly contribute to their different performance under freezing tolerance.

In roots, *PnDREB1* exhibits higher expression levels than those of other tissues under normal condition. However, in mangrove *Aegiceras corniculatum*, the highest expression was detected in leaves (Peng *et al.*, 2015). This suggests that DREB1 may function diversely in plant development in different species. Under freezing stress, *PnDREB1* was induced gradually and exhibited first a down- and then up-regulated pattern, which seems to be complementary to that in leaves (Figures 5b and c). Few reports individually addressed the expression changes in roots under stress. However, we speculated that this might be due to two reasons: first, our freezing treatment was performed under hydroponic condition, by which the leaves might perceive freezing stress more quickly than roots; second, there might exist a balance *PnDREB1* expression between roots and leaves.

ABA is an important plant hormone that plays a regulatory role in many development processes in plants, as well as in the activation of stress-responsive genes (Agarwal and Jha, 2010). Previous studies in *Arabidopsis* showed that *DREB1D/CBF4* is rapidly induced by drought and ABA but not by cold stress (Haake *et al.*, 2002), whereas *DREB1B/CBF1, DREB1A/ CBF3*, and *DREB1C/ CBF2* are strongly and transiently induced by low temperature stresses but not by ABA or dehydration (Gilmour *et al.*, 1998; Medina *et al.*, 1999). However, these different results come from diverse plant species. *PNDREB1* of *Arachis hypogaea* was strongly upregulated by treatments with low temperature, and also responded to dehydration (Zhang *et al.*, 2009); *PpDBF1* of *Physcomitrella patens* was simultaneously induced by NaCl, cold, drought, and ABA (Liu *et al.*, 2007). The results obtained in this study showed that besides freezing treatment, *PnDREB1* is also rapidly induced by ABA, especially in roots, suggesting that *PnDREB1* is possibly involved in other abiotic stress responses, such as drought and NaCl. Further research is needed to clarify this speculation.

## Supplementary material

The following online material is available for this article:

Figure S1 - Nucleotide and deduced amino acid sequences of *actin1* and *EF1.*

Figure S2 - Melting curves.

Figure S3 - Cloning of DREB1 from Iceland poppy.

Figure S4 - Motif logos in 36 DREB1s.

Table S1 - Primer sequences.
