# Supplementary material for: Molecular cloning and characterization of a novel freezing-inducible DREB1/CBF transcription factor gene in boreal plant Iceland poppy (Papaver nudicaule)
Source: Genet Mol Biol. 2016 Jul 25;39(4):616–28. doi: 10.1590/1678-4685-GMB-2015-0228 (PMC5127145; doi:10.1590/1678-4685-GMB-2015-0228)
Supplement: Supplementary file 1 [file 1415-4757-gmb-1678-4685-GMB-2015-0228-Suppl05.pdf]

Table S1 - Primer sequences used for gene cloning

Primer Name	Sequence (5'-3')	Target Gene	Expected Size (bp)	Usage
Oligo adapter	AAGCAGTGGTATCAACGCAGAGTACTTTTTTTTTTTTTTTTTTTT			reverse transcription
DREB1-F1	GAGACRCGRCAYCCRRTBTAYAG	PnDREB1	~200	Cloning
DREB1-R1	CCGCCAWGCMGARTCWGSRAART			
3UPM	AAGCAGTGGTATCAACGCAGAGT	PnDREB1	~770	3'RACE
3'RACE-GSP1(outer)	TAGAGGAGTGAGGCAAAGGAACAA			
3'RACE-GSP2(iner)	GGGTATGTGAAGTAAGAGAAC			
5'RACE-GSP1(outer)	AACAGGTACAGGCAATCTCCAGAC	PnDREB1	~470	5'RACE
5'RACE-GSP2(inner)	GGAATCAGCGAAATTAAGACAAGC			
5'RACE Outer Primer	CATGGCTACATGCTGACAGCCTA			
5'RACE Inner Primer	CGCGGATCCACAGCCTACTGATGATCAGTCGATG			
ef1F1	GGAGGTATTGACAAGCGTGT	*Elogantion Factor 1*	~500	Cloning
ef1R1	AGGGTTGGACCCTTGTACCA			
actin-F1	AATGGAACTGGAATGGTGAAG	*Actin1*	~800	CLoning
actin-R1	TATCAACATCACACTTCATGAT-3			

Note: R(A/G), Y(C/T), B(C/G/T), W(A/T), M(A/C),S(C/G)
