# Supplementary material for: Molecular cloning and characterization of a novel freezing-inducible DREB1/CBF transcription factor gene in boreal plant Iceland poppy (Papaver nudicaule)
Source: Genet Mol Biol. 2016 Jul 25;39(4):616–28. doi: 10.1590/1678-4685-GMB-2015-0228 (PMC5127145; doi:10.1590/1678-4685-GMB-2015-0228)
Supplement: Supplementary file 2 [file 1415-4757-gmb-1678-4685-GMB-2015-0228-Suppl01.pdf]

a

```
GGAACTGGAATGGTGAAGGCCGGATTCGCTGGAGATGATGCTCCAAGGGCTGTGTTCCCTAGTATTGTGGGTCGCCCTCGCCACACTGGT 90
 G  T  G  M  V  K  A  G  F  A  G  D  D  A  P  R  A  V  F  P  S  I  V  G  R  P  R  H  T  G

GTGATGGTTGGTATGGGTCAGAAAGATGCGTATGTTGGGGATGAAGCTCAATCAAAGAGAGGTATCTTAACTTTGAAATACCCAATTGAG 180
 V  M  V  G  M  G  Q  K  D  A  Y  V  G  D  E  A  Q  S  K  R  G  I  L  T  L  K  Y  P  I  E

CATGGTATTGTGAGCAACTGGGATGACATGGAGAAAATCTGGCATCACACTTTCTACAATGAACTCCGTGTGGCCCCAGAAGAGCACCCT 270
 H  G  I  V  S  N  W  D  D  M  E  K  I  W  H  H  T  F  Y  N  E  L  R  V  A  P  E  E  H  P

GTTCTCCTCACTGAAGCACCTCTCAACCCAAAGGCTAATCGTGAGAAGATGACTCAAATCATGTTTGAAACCTTCAACACTCCTGCCATG 360
 V  L  L  T  E  A  P  L  N  P  K  A  N  R  E  K  M  T  Q  I  M  F  E  T  F  N  T  P  A  M

TATGTTGCCATCCAGGCTGTTCTGTCCCTATATGCCAGTGGTCGTACTACTGGTATTGTCTTGGATTCCGGGGATGGTGTCAGCCATACA 450
 Y  V  A  I  Q  A  V  L  S  L  Y  A  S  G  R  T  T  G  I  V  L  D  S  G  D  G  V  S  H  T

GTTCCCATCTATGAAGGGTATGCCCTACCACATGCCATCCTGCGTCTTGACCTGGCAGGACGTGATCTCACTGATGCATTGATGAAAATC 540
 V  P  I  Y  E  G  Y  A  L  P  H  A  I  L  R  L  D  L  A  G  R  D  L  T  D  A  L  M  K  I

TTGACGGAGCGTGGTTACTCTTTCACCACCACAGCCGAGCGGGAAATTGTGAGAGACATGAAGGAAAAGCTAGCTTACATTGCTCTTGAT 630
 L  T  E  R  G  Y  S  F  T  T  T  A  E  R  E  I  V  R  D  M  K  E  K  L  A  Y  I  A  L  D

TATGAGCAAGAGCTAGAGACATCCAAGACCAGTTCTTCTGTTGAAAAGAGCTATGAGTTACCTGATGGACAAGTAATCACCATTGGAGCT 720
 Y  E  Q  E  L  E  T  S  K  T  S  S  S  V  E  K  S  Y  E  L  P  D  G  Q  V  I  T  I  G  A

GAACGTTTCCGTTGCCCTGAAGTTCTTTTCCAGCCATCAATGATCGGTATGGAAGCTGCTGGAATTCACGAGACTACATACAACTCTATC 810
 E  R  F  R  C  P  E  V  L  F  Q  P  S  M  I  G  M  E  A  A  G  I  H  E  T  T  Y  N  S  I

ATGAAGTGTGATGTTGATA 829
 M  K  C  D  V  D
```

b

```
GGAGGTATTGACAAGCGTGTGATTGAGAGGTTCGAGAAGGAGGCTGCTGAGATGAACAAGAGGTCTTTCAAGTATGCATGGGTTCTAGAC 90
 G  G  I  D  K  R  V  I  E  R  F  E  K  E  A  A  E  M  N  K  R  S  F  K  Y  A  W  V  L  D

AAGCTTAAGGCTGAACGTGAGCGTGGTATTACTATTGATATTGCCTTGTGGAAGTTTGAGACCACCAAGTACTACTGCACAGTCATTGAT 180
 K  L  K  A  E  R  E  R  G  I  T  I  D  I  A  L  W  K  F  E  T  T  K  Y  Y  C  T  V  I  D

GCTCCAGGACACCGTGATTTCATCAAGAACATGATTACTGGTACCTCTCAGGCTGATTGTGCTGTCCTCATTATTGACTCCACCACTGGA 270
 A  P  G  H  R  D  F  I  K  N  M  I  T  G  T  S  Q  A  D  C  A  V  L  I  I  D  S  T  T  G

GGTTTTGAGGCCGGAATATCCAAGGATGGACAGACTCGTGAGCACGCTCTTCTTGCTTTTACTCTTGGTGTCAAGCAGATGATCTGTTGC 360
 G  F  E  A  G  I  S  K  D  G  Q  T  R  E  H  A  L  L  A  F  T  L  G  V  K  Q  M  I  C  C

TGTAACAAGATGGATGCCACCACGCCCAAGTACTCCAAGGCTAGGTACGATGAAATTGTCAAGGAGGTCTCATCTTACTTGAAGAAGGTT 450
 C  N  K  M  D  A  T  T  P  K  Y  S  K  A  R  Y  D  E  I  V  K  E  V  S  S  Y  L  K  K  V

GGGTACAACTCAGACAAAATTGCCTTTGTTCCCATCTCTGGATTTGAGGGAGATAACATGATTGAGAGGTCCACAAACCTTGACTGGTAC 540
 G  Y  N  S  D  K  I  A  F  V  P  I  S  G  F  E  G  D  N  M  I  E  R  S  T  N  L  D  W  Y

AAGGGTCCAACCCT 554
 K  G  P  T
```

Figure S1 - Partial nucleotide and deduced amino acid sequences of *actin1* (a) and *EF1* (b) obtained in this study.
