# Supplementary figures and images for: Molecular cloning and characterization of a novel freezing-inducible DREB1/CBF transcription factor gene in boreal plant Iceland poppy (Papaver nudicaule)
Source: Genet Mol Biol. 2016 Jul 25;39(4):616–28. doi: 10.1590/1678-4685-GMB-2015-0228 (PMC5127145; doi:10.1590/1678-4685-GMB-2015-0228)

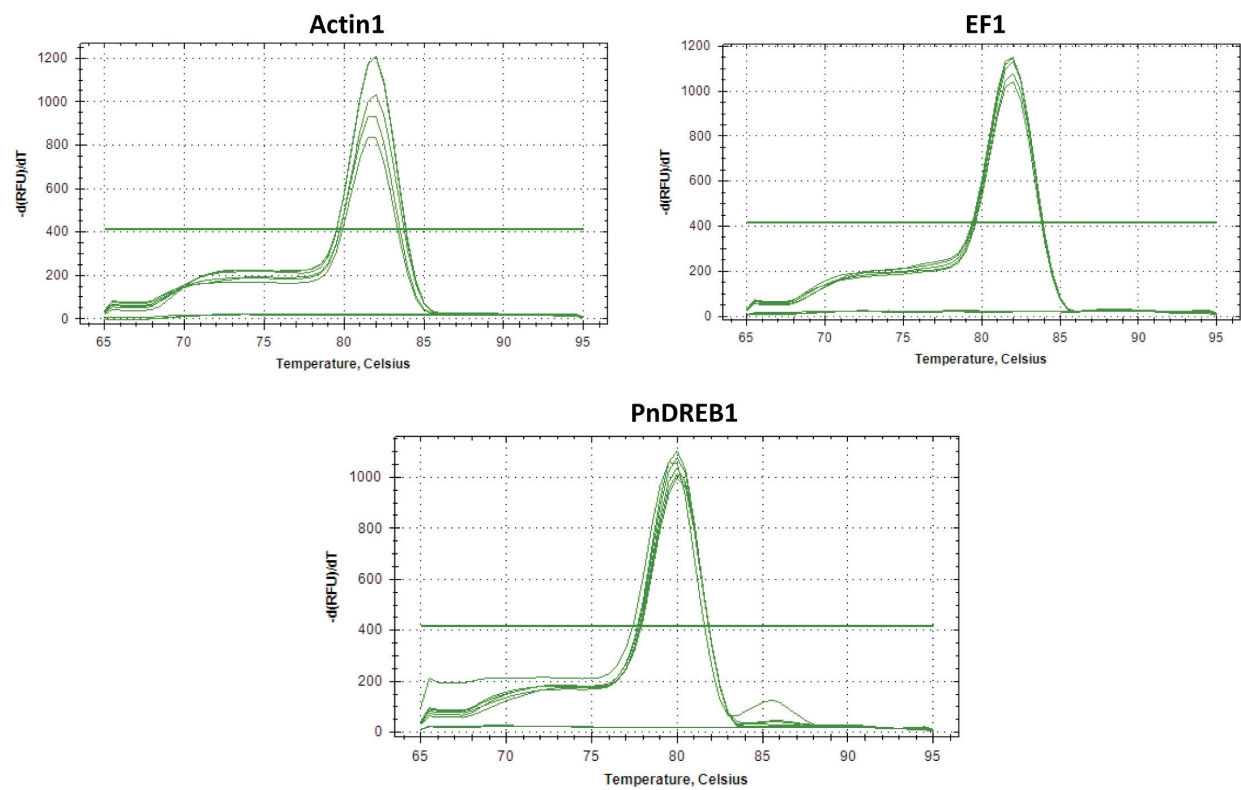

**Figure S2** - Melting curve of the targeting gene primers and the internal controls.

Supplement: Supplementary file 3 [file 1415-4757-gmb-1678-4685-GMB-2015-0228-Suppl02.pdf]

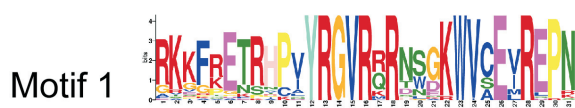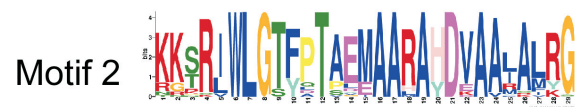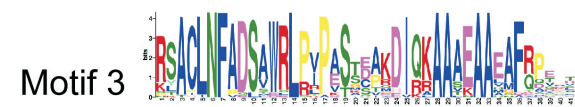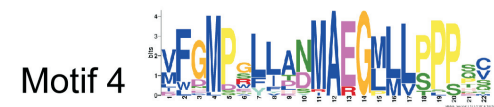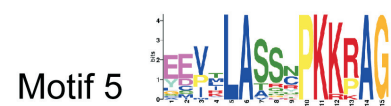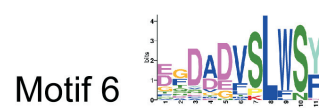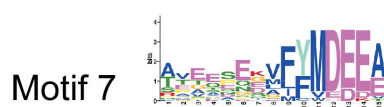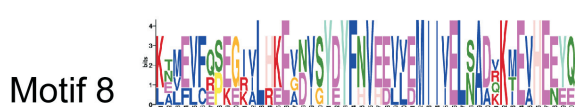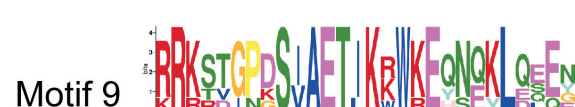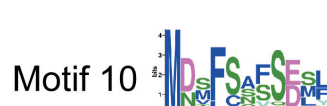

**Figure S4 - Motif logos of ten motifs identified in 36 DREB1s.**

Supplement: Supplementary file 5 [file 1415-4757-gmb-1678-4685-GMB-2015-0228-Suppl04.pdf]
