# Supplementary material for: Molecular cloning and characterization of a novel freezing-inducible DREB1/CBF transcription factor gene in boreal plant Iceland poppy (Papaver nudicaule)
Source: Genet Mol Biol. 2016 Jul 25;39(4):616–28. doi: 10.1590/1678-4685-GMB-2015-0228 (PMC5127145; doi:10.1590/1678-4685-GMB-2015-0228)
Supplement: Supplementary file 4 [file 1415-4757-gmb-1678-4685-GMB-2015-0228-Suppl03.pdf]

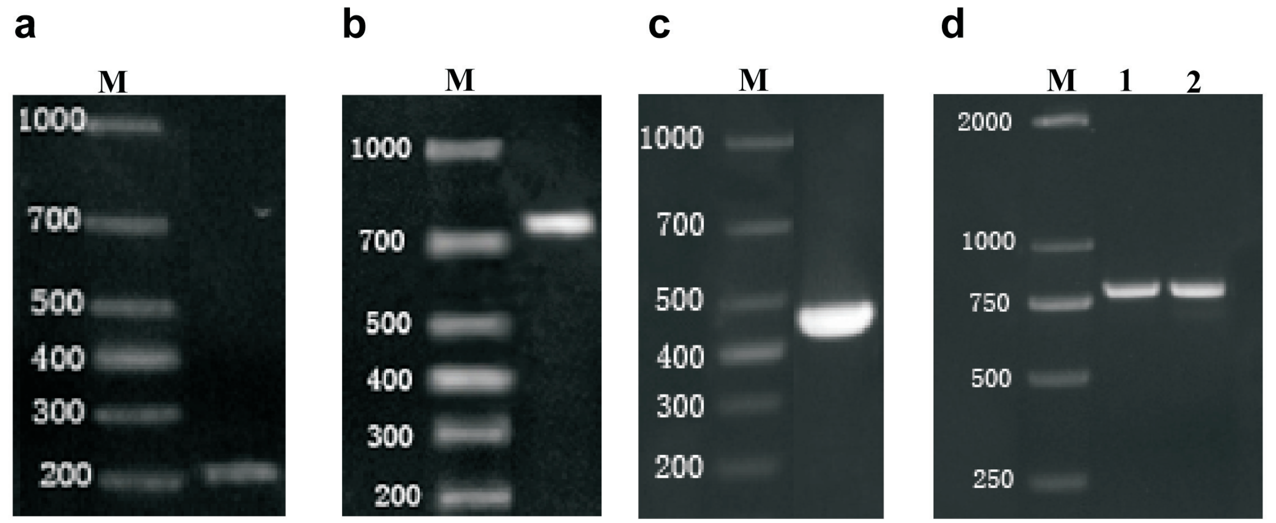

Figure S3 - Cloning of DREB1 from Iceland poppy. a, amplification of conserved AP2/ERF domain by RT-PCR; b, Fragment amplified by 3'RACE; c, Fragment amplified by 5' RACE; d, Validation by RT-PCR (1) and genomic PCR (2). M indicates DNA ladder and numbers on the left column of each image show length (base pairs) of the fragment.
